# Supplementary material for: Francisella requires dynamic type VI secretion system and ClpB to deliver effectors for phagosomal escape
Source: Nat Commun. 2017 Jun 16;8:15853. doi: 10.1038/ncomms15853 (PMC5481754; doi:10.1038/ncomms15853)
Supplement: Supplementary Information [file ncomms15853-s1.pdf]

Type of file: PDF
Size of file: 0 KB
Title of file for HTML: Supplementary Information
Description: Supplementary Figures and Supplementary Tables

Type of file: MP4
Size of file: 0 KB
Title of file for HTML: Supplementary Movie 1
Description: T6SS sheath dynamics in wild-type and mutant strains. IglAsfGFP was monitored in F. novicida U112 iglA-sfGFP wild-type, ΔpdpE, ΔanmK, ΔpdpC, ΔpdpD, ΔpdpD/anmK, ΔpdpC/pdpD, ΔpdpC/pdpD/anmK and ΔclpB for 5 min at a frame rate of 20 frames per minute. 2 representative time-lapse image series for each strain are shown. Merge of phase contrast and GFP channel is shown. Fields of view are 39 x 26 µm. Videos play at a frame rate of 10 frames per second.

Type of file: MP4
Size of file: 0 KB
Title of file for HTML: Supplementary Movie 2
Description: Knockout of critical components abolishes T6SS dynamics. IglAsfGFP was monitored in F. novicida U112 iglA-sfGFP ΔpdpB, ΔclpB/pdpB, ΔiglF, ΔiglG, ΔiglI and ΔiglJ for 5 min at a frame rate of 2 frames per minute. 2 representative time-lapse image series for each strain are shown. Merge of phase contrast and GFP channel is shown. Fields of view are 39 x 26 µm. Videos play at a frame rate of 5 frames per second.

Type of file: MP4
Size of file: 0 KB
Title of file for HTML: Supplementary Movie 3
Description: ClpB spots co-localize with contracted sheaths. IglA-sfGFP and ClpB-mCherry2 was monitored in F. novicida U112 iglA-sfGFP clpB-mCherry2, iglA-sfGFP clpBmCherry2 ΔpdpB and clpB-mCherry2 for 5 min at a frame rate of 2 frames per minute. 2 representative time-lapse image series for each strain are shown. Merge of phase contrast, GFP and mCherry channels is shown. Fields of view are 39 x 26 µm. Videos play at a frame rate of 5 frames per second.

Type of file: MP4
Size of file: 0 KB
Title of file for HTML: Supplementary Movie 4
Description: Examples of wild-type and ΔclpB T6SS sheath dynamics. IglAsfGFP was monitored in F. novicida U112 iglA-sfGFP wild-type and ΔclpB for 5 min at a frame rate of 20 frames per minute. 10 representative time-lapse image series for each strain are shown. GFP channel is shown. Fields of view are 3.3 x 3.3 µm. Videos play at a frame rate of 10 frames per second.

Type of file: MP4
Size of file: 0 KB
Title of file for HTML: Supplementary Movie 5
Description: Examples of co-localization of ClpB with contracted sheath. IglAsfGFP and ClpB-mCherry2 was monitored in F. novicida U112 iglA-sfGFP clpB-mCherry2 for 5 min at a frame rate of 20 frames per minute. 10 representative time-lapse image series are shown. Left fields show GFP channel and right fields show mCherry channel. Fields of view are

3.3 x 3.3 µm. Videos play at a frame rate of 10 frames per second.

Type of file: MP4
Size of file: 0 KB
Title of file for HTML: Supplementary Movie 6
Description: Examples of T6SS dynamics in infected bone marrow derived macrophages. IglA-sfGFP and ClpB-mCherry2 was monitored in F. novicida U112 iglA-sfGFP clpB-mCherry2 for 10 min at a frame rate of 2 frames per minute. 5 representative time-lapse image series are shown. Left fields show phase contrast channel, middle fields show GFP channel and right fields show mCherry channel. Fields of view are 30 x 30 µm. Videos play at a frame rate of 5 frames per second.

Type of file: PDF
Size of file: 0 KB
Title of file for HTML: Peer Review File
Description:

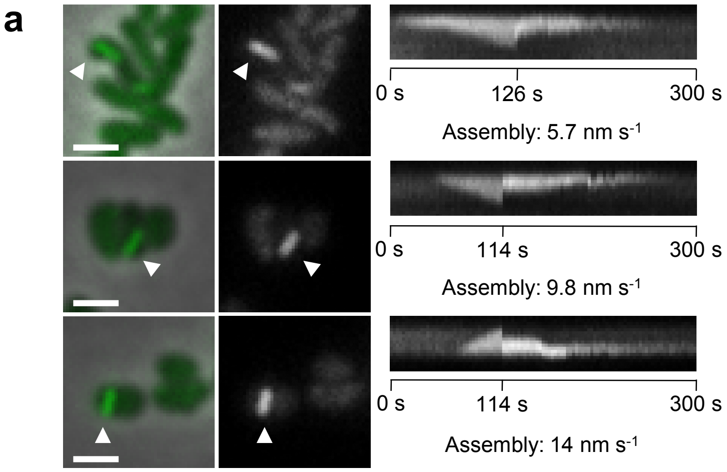

b

$$\frac{GFP\ intensity_{\text{Before assembly}}}{GFP\ intensity_{\text{After assembly}}} = 1.06 \pm 0.07$$

$$\frac{GFP\ intensity_{F.novicida\ \text{U112}-sfGFP\ \Delta pdpB}}{GFP\ intensity_{F.novicida\ \text{U112}-sfGFP}} = 0.96 \pm 0.23$$

Supplementary Figure 1 : Assembly speed varies between bacteria. (a) Kymograms of slow (~ 5 nm s^{-1}) to fast (~ 14 nm s^{-1}) T6SS assemblies (arrowheads) over 5 minutes (3 s per pixel) in *F. novicida* U112 *iglA-sfGFP*. First image is a merge of phase contrast and GFP channel, following images represent GFP channel only. 3.3 x 3.3 µm fields of view are shown. Scale bars represent 1 µm. **(b)** GFP intensities were measured a frame before and a frame after a complete assembly-disassembly cycle in two independent experiments. 30 bacteria were analyzed per experiment. GFP intensities measured in *F. novicida* U112 *iglA-sfGFP* wild-type *and ΔpdpB* were compared in four independent experiments. 30 bacteria were analyzed per experiment. Standard deviation was calculated.

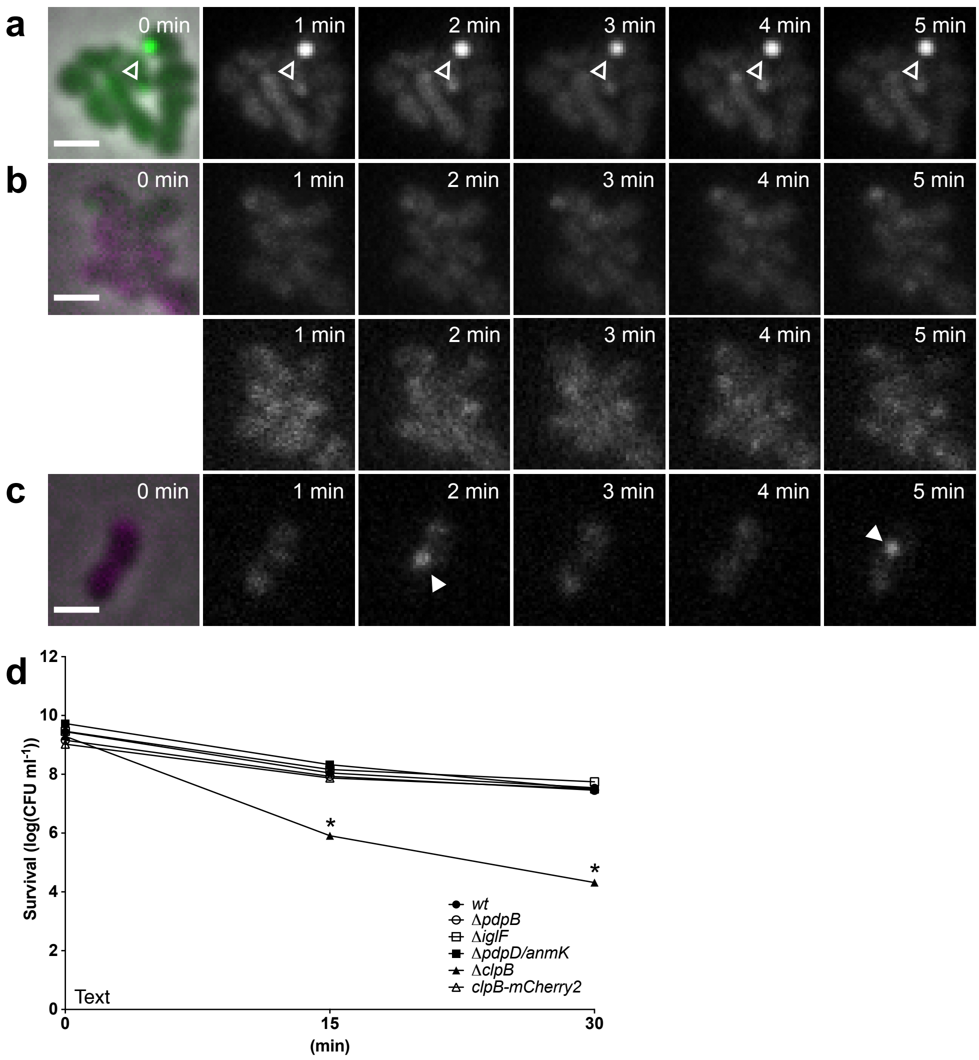

Supplementary Figure 2: T6SS activity is required for ClpB spot localization but dispensable for ClpB-dependent heat tolerance. (a) IglA-sfGFP localization and foci (empty arrowheads) in *F. novicida U112 iglA-sfGFP ΔclpB/pdpB*. First image is a merge of phase contrast and GFP channels, following images represent GFP channel only. 3.3 x 3.3 µm fields of view are shown. Scale bars represent 1 µm. **(b)** IglA-sfGFP and ClpB-mCherry2 localization in *F. novicida* U112 *iglA-sfGFP clpB-mCherry2 ΔpdpB*. First image is a merge of phase contrast, GFP and mCherry channels, following images represent GFP channel (upper panel) and mCherry channel (lower panel). **(c)** ClpB-mCherry2 localization dynamics in *F. novicida* U112 *clpB-mCherry2*. First image is a merge of phase contrast and mCherry channels, following images represent mCherry channel only. Arrowheads indicate ClpB recruitment. **(d)** Heat shock survival assay performed with *F. novicida U112 iglA-sfGFP* wild-type, *ΔpdpB*, *ΔiglF*, *ΔpdpD/anmK*, *ΔclpB* and *clpB-mCherry2* at 50 °C for 0, 15 and 30 min. Data are pooled from three independent experiments. $^*P < 0.05$ (two-tailed unpaired *t*-test with Welch's correction). (a-c) 3.3 x 3.3 µm fields of view are shown. Scale bar represents 1 µm.

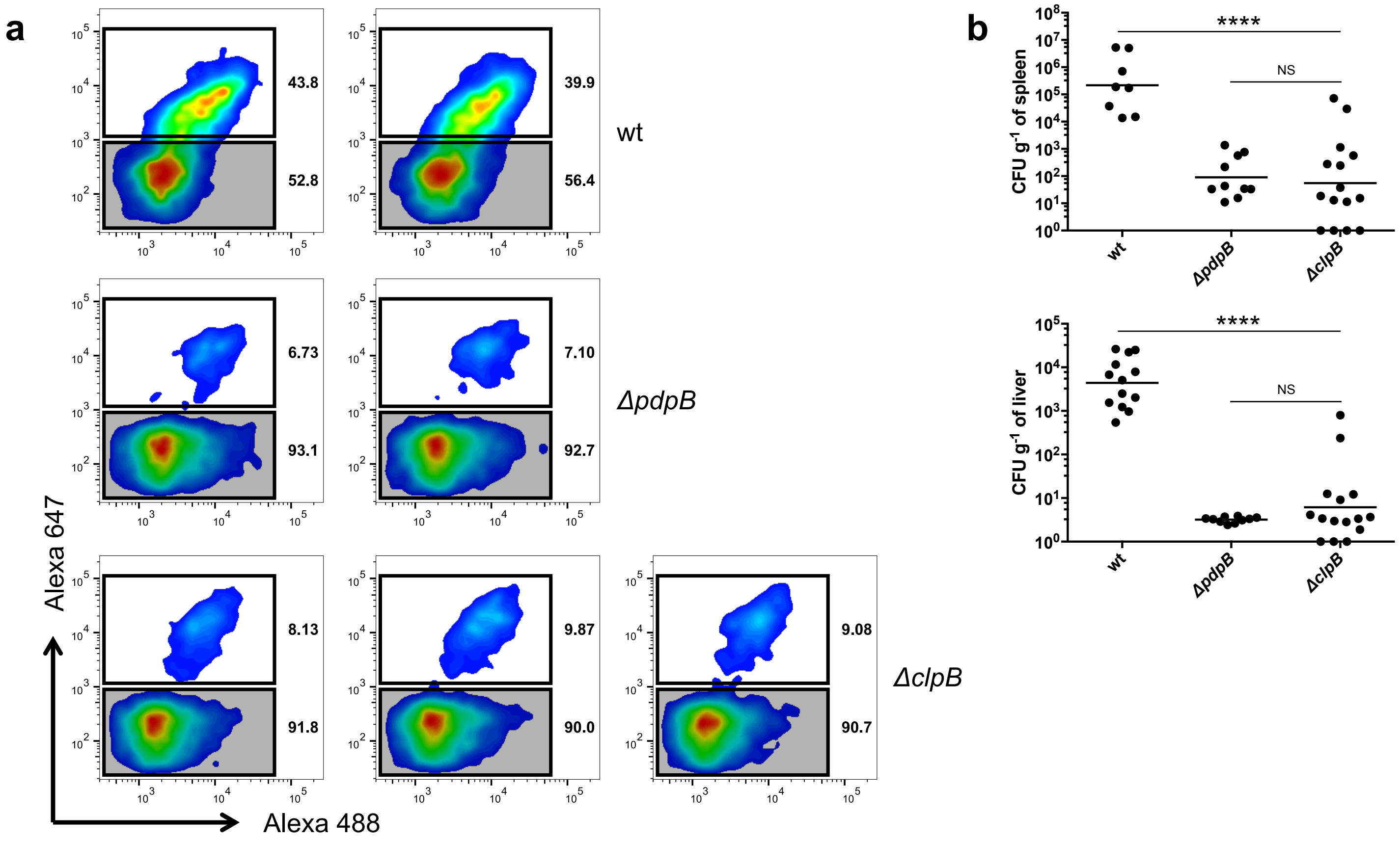

Supplementary Figure 3: *F. novicida* U112 *iglA-sfGFP ΔclpB* fails to escape into the cytosol and is avirulent *in vivo*. **(a)** Representative FACS blots from the quantification of cytosolic (white gates) and vacuolar bacteria (grey gates) by flow cytometry in unprimed wild-type BMDMs 4 h after infection with *F. novicida* U112 *iglA-sfGFP* wild-type*, ΔpdpB* or *ΔclpB.* Numbers next to the gates indicate the percentage of cytosolic and vacuolar bacteria. **(b)** Bacterial burden (as colony-forming units (CFU) per gram tissue) in the spleen and liver of wild-type C57BL/6JRj mice infected subcutaneously for 2 days with 1 x 10^4 *F. novicida* U112 *iglA-sfGFP* wild-type*, ΔpdpB* or *ΔclpB.* Each symbol represents an individual mouse (n = 8 (wild-type), 10 (*ΔpdpB)*, 15 (*ΔclpB*) (spleen), or n = 13 (wild-type), 10 (*ΔpdpB),* 15 (*ΔclpB*) (liver)); small horizontal lines indicate the mean. Data are pooled from two independent experiments. ****$P < 0.0001$; NS - not significant (Mann-Whitney test).

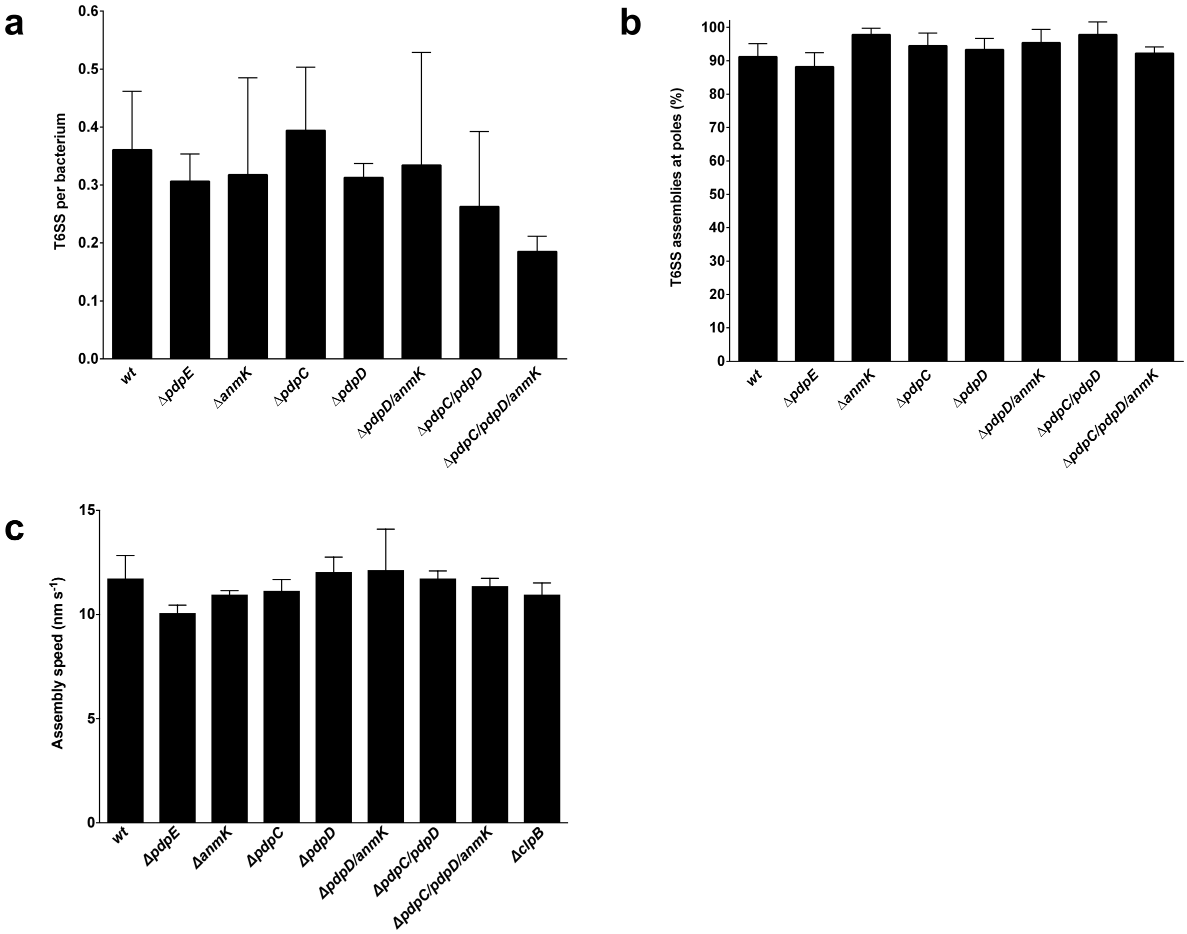

Supplementary Figure 4: *pdpE*, *anmK, pdpC* and *pdpD* and play no role in T6SS sheath localization and dynamics. (a) Quantification of number of T6SS sheath structures per bacterium within 5 min of imaging. **(b)** Quantification of T6SS sheath assembly at poles. **(c)** Quantification of T6SS assembly speed. Averages of three independent experiments. 30 bacteria per experiment were analyzed. Error bars represent standard deviation. No significant differences to wild-type (two-tailed unpaired *t*-test with Welch's correction).

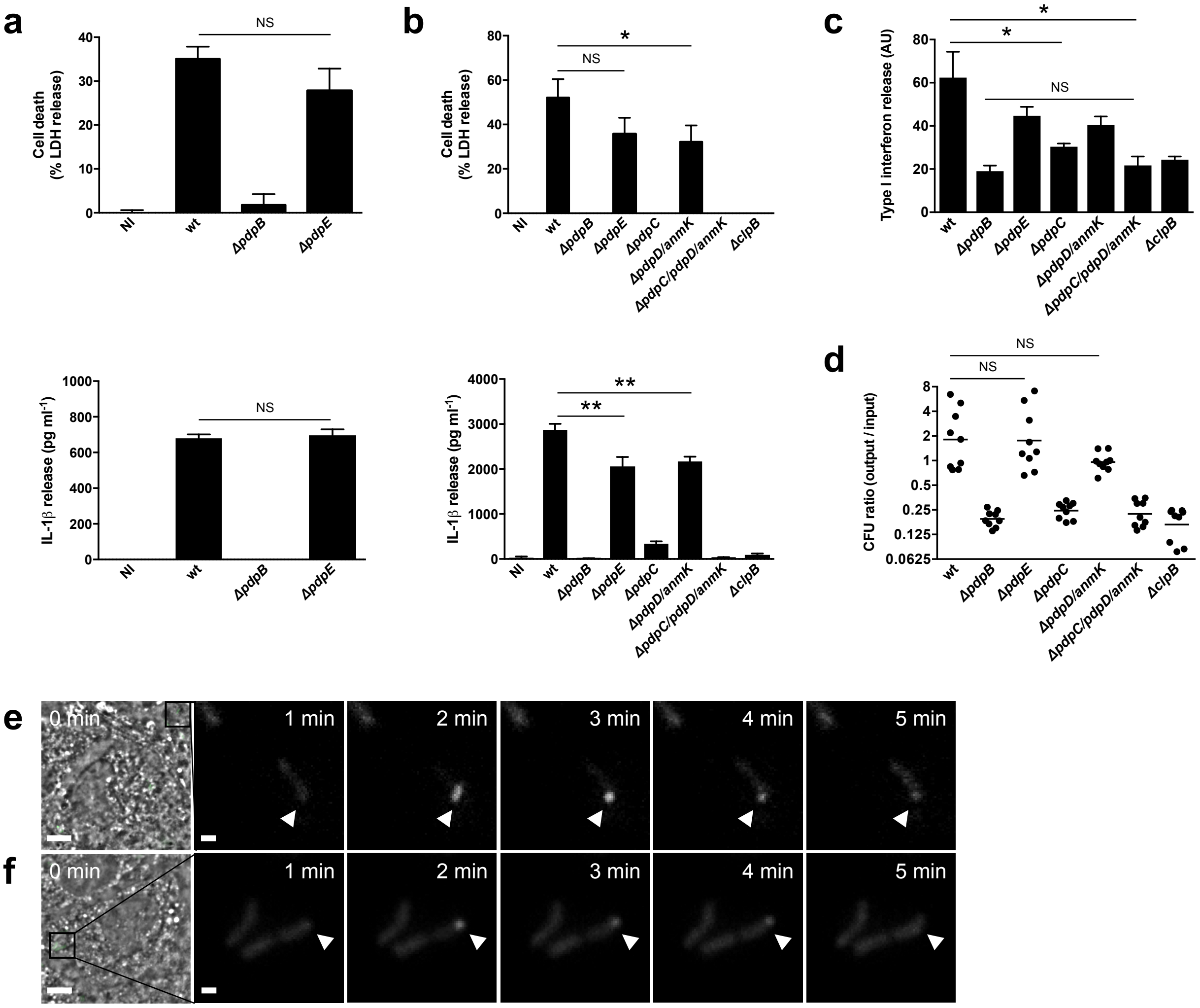

Supplementary Figure 5: Putative effector mutants show distinct innate immune activation and survival within macrophages. Release of LDH and IL-1β from **(a)** LPS-primed wild-type BMDMs 10 h or **(b)** unprimed wild-type BMDMs 24 h after infection with *F. novicida* U112 *iglA-sfGFP* wild-type, *ΔpdpB, ΔpdpE, ΔpdpC, ΔpdpD, ΔpdpD/anmK, ΔpdpC/pdpD/anmK* or *ΔclpB* (NI - noninfected control). **(c)** Quantification of type-I-interferon release in the supernatant of unprimed wild-type BMDMs infected for 10 h with *F. novicida* U112 *iglA-sfGFP* wild-type, *ΔpdpB, ΔpdpE, ΔpdpC, ΔpdpD, ΔpdpD/anmK, ΔpdpC/pdpD/anmK* or *ΔclpB.* **(d)** Intracellular growth within *Asc*$^{-/-}$ BMDMs during the first 24 h of infection with *F. novicida* U112 *iglA-sfGFP* wild-type, *ΔpdpB, ΔpdpE, ΔpdpC, ΔpdpD/anmK, ΔpdpC/pdpD/anmK* or *ΔclpB.* Growth was calculated as ratio of number of bacteria at 24 h (output) divided by the number of bacteria at 2 h (input). **(f, e**) Timelapse images from BMDMs infected for 1 h with *F. novicida* U112 *iglA-sfGFP ΔpdpE* **(e)** and *ΔpdpC/ pdpD/anmK* **(f)**. 30 x 30 µm fields of view are shown. First image consists of merged phase contrast channel and GFP channel. Scale bar represents 5 µm. The close ups show 5 x 5 µm. Scale bar represents 1 µm. Close ups consist of GFP channel. (a-d) Data are representatives of three independent experiments (a-c) (mean and standard deviation of triplicate wells are shown) or pooled from three independent experiments (small horizontal lines indicate the mean) (d). *$*P < 0.05$ and **$*P < 0.01$; NS - not significant (two-tailed unpaired *t*-test with Welch's correction).

Supplementary Table 1: Strains used in this study, related to Material and Methods

Organism	Genotype	Relevant features	Source
Francisella novicida U112	*iglA-sfGFP*	Parental strain, C-terminal chromosomal fusion of *sfGFP* to *iglA*	(Clemens et al., 2015)
	iglA-sfGFP ΔpdpB	Deletion of *pdpB*	This study
	iglA-sfGFP ΔclpB	Deletion of *clpB*	This study
	iglA-sfGFP ΔclpB/pdpB	Deletion of *clpB* and *pdpB*	This study
	iglA-sfGFP clpB-mCherry2	C-terminal chromosomal fusion of *mCherry2* to *clpB*	This study
	iglA-sfGFP clpB-mCherry2 ΔpdpB	C-terminal chromosomal fusion of *mCherry2* to *clpB,* deletion of *pdpB*	This study
	clpB-mCherry2	C-terminal chromosomal fusion of *mCherry2* to *clpB*	This study
	iglA-sfGFP ΔiglF	Deletion of *iglF*	This study
	iglA-sfGFP ΔiglG	Deletion of *iglG*	This study
	iglA-sfGFP ΔiglI	Deletion of *iglI*	This study
	iglA-sfGFP ΔiglJ	Deletion of *iglJ*	This study

iglA-sfGFP ΔpdpE	Deletion of *pdpE*	This study
iglA-sfGFP ΔanmK	Deletion of *anmK*	This study
iglA-sfGFP ΔpdpC	Deletion of *pdpC*	This study
iglA-sfGFP ΔpdpD	Deletion of *pdpD*	This study
iglA-sfGFP ΔpdpD/anmK	Deletion of *pdpD* and *anmK*	This study
iglA-sfGFP ΔpdpC/pdpD	Deletion of *pdpC* and *pdpD*	This study
iglA-sfGFP ΔpdpC/pdpD/anmK	Deletion of *pdpC*, *pdpD* and *anmK*	This study

Supplementary Table 2: Plasmids used to generate in-frame deletions in this study, related to Material and Methods

Plasmid Name	Peptide scar left on the chromosome after allelic exchange	Primers used to generate in-frame deletion	
pDMK3-Δ*pdpB*	MNFIQKQGEVNVQ*	dFTN_1310_Del1_Xho1.FOR	TCAGTACTCGAGCAACTATATGAAAACTTACATAATT
		dFTN_1310_Del1.REV	CTCCTTGTTTTTGAATAAAATTCATACTTTTAATTT
		dFTN_1310_Del2_.FOR	ATGAATTTTATTCAAAAACAAGGAGAAGTTAATGT
		dFTN_1310_Del2.REV	ATAATAGCGGCCGCTTAGCAGAGCTTTTTATATT
		dFTN_1310_Det_FOR	ACATCAAGAAATACTCTGCCCTTC
		dFTN_1310_Det_REV	TATTATTATCCAACCATTGTTGCTG
pDMK3-Δ*clpB*	MNINKFTIKLANNNITFSK*	dFTN_1743_1_Spe1.FOR	TCAGTAACTAGTAGATAAATGCGACTATTGATG
		dFTN_1743_1.REV	TAATATTGTTATTAGCTAGTTTTATTGTAAATTTATTTATATTCATTATTT

	dFTN_1743_2.FOR	ATTTACAATAAAACTAGCTAATAACAATATTACATTCTCTAAA
	dFTN_1743_2_Sac1.REV	TCAGTAGAGCTCTCTTTTGTCATTGCAAAAGA
	dFTN_1743_Det.FOR	CAAGAATTCCATCAACCCAGA
	FTN_1743-mCherry_Det_REV	CCATCAAACTTAACAAAAGCTCCT
pDMK3-*clpB-mCherry2*	FTN_1743-mcherry1_Spe1.FOR	TCAGTAACTAGTGGTGTCGGTAAAACTGA
	FTN_1743-mcherry1.REV	CGGCCGCTTTAGAGAATGTAATATTGTTATTAGCG
	FTN_1743-mcherry2.FOR	CTCTAAAGCGGCCGCAGGA
	FTN_1743-mcherry2.REV	ATTAAACCGATTTTACTTGTACAGCTCGTC
	FTN_1743-mcherry3.FOR	CTGTACAAGTAAAATCGGTTTAATCAATATCTAAATTAT
	FTN_1743-mcherry3_Sac1.REV	TCAGTAGAGCTCGCTTTATAAGTTAGATTAATAGAGTTTG
	FTN_1743-mcherry_Det_FOR	GATGGAAGGCGAAAAAGACA
	FTN_1743-mcherry_Det_REV	CCATCAAACTTAACAAAAGCTCCT

pDMK3-∆*iglF*	MNNDIDKWFESKQEAYWKI*	dFTN_1313_1_Spe1.FOR	TCAGTAACTAGTTTTCTCAAAGAATATATGATGATAATG
		dFTN_1313_1.REV	TTGCTTGCTTTCAAACCATTTATCAATATCATTATT
		dFTN_1313_2.FOR	TGGTTTGAAAGCAAGCAAGAAGC
		dFTN_1313_2_Sac1.REV	TCAGTAGAGCTCTATTTCTAATAAGCATGATTTA GGAA
		dFTN1313_Det.FOR	CTGGGTAATCAAGCACAAAGGT
		dFTN1313_Det.REV	GTGGCAAAGCTAGGATCTTCT
pDMK3-∆*iglG*	MLNIINDSLKGGQINVKTS*	dFTN_1314_1_Xho1.FOR	TCAGTACTCGAGATAAAAAATCAACTCTACAAAAACC
		dFTN_1314_1.REV	TTTGTCCACCTTTTAAGGAGTCATTTATAATATTTAACATT
		dFTN_1314_2.FOR	CTCCTTAAAAGGTGGACAAATAAATGTAAA
		dFTN_1314_2_Not1.REV	TCAGTAGCGGCCGCTAATTTTTCGTCATTATAGTTTTCAG
		dFTN_1314_Det.FOR	TTTCGCTAACGTCACTACAAGC
		dFTN_1314_Det.REV	TCATCGAAGCAAATGAGGTG

pDMK3-Δ*iglI*	MSQIISTLNNDSVEKISNEIDED YFEDLFDI*	dFTN_1317_1_Xho1.FOR	TCAGTACTCGAGAAATTTATAAATCAAAACACCTTTAGC
		dFTN_1317_1.REV	TTCTACCGAATCATTATTTAGTGTAGATATTATCTGACT
		dFTN_1317_2.FOR	ACACTAAATAATGATTCGGTAGAAAAAATTT
		dFTN_1317_2_NotI.REV	TCAGTAGCGGCCGCATTTCAAGTTCTATCTTAAATGGG
		dFTN_1317_Det.FOR	ATCGCAGCACACAATCTTTAAA
		dFTN_1317_Det.REV	TCAGATAGTGATTCGGATTTTTCA
pDMK3-Δ*iglJ*	MKTILKIFLTYKQQIYLGYFNL*	dFTN_1318_1_Xho1.FOR	TCAGTACTCGAGATAACATAGATTCTATTATAGAAATTGTACA
		dFTN_1318_1.REV	CCTAGATATATCTGTTGTTTATATGTCAAAAAGATCTTCAAA
		dFTN_1318_2.FOR	GATCTTTTTGACACAGATATATCTAGGTTATTTTAATTTATG
		dFTN_1318_2_NotI.REV	TCAGTAGCGGCCGCATCATTTGCGCTTATTTCAA
		dFTN_1318_Det.For	CGCAAATGCAGAATCAAGAA
		dFTN_1318_Det.Rev	CGACTAGCGCGTCTAAAAATG
pDMK3-Δ*pdpE*	MSKKVFQLLLHYEKKITII*	dFTN_1320_1_Xho1.FOR	TCAGTACTCGAGACCAACAGAAGAAAACTTTG
		dFTN_1320_1.REV	ATTTTCTTTTCATAATGTAATAATAATTGAAATACTTTTTTACTCATATT

		dFTN_1320_2.FOR	ATTTCAATTATTATTACATTATGAAAAGAAAATTACTATAATATAAC
		dFTN_1320_2_Not1.REV	TCAGTAGCGGCCGCGGTGATATTTTTGTAAAACTTAATAGG
		dFTN_1320_Det.FOR	GGGTTGGGCTATCACATCAA
		dFTN_1320_Det.REV	GTTGAAAGTTTGCAGACAGGTC
pDMK3-Δ*anmK*	LSEYKYCVGIIPSATGAKSRVILGQINFF*	dFTN_1326_1_Xho1.FOR	TCAGTACTCGAGCTTAGGTATAATGGAATAAATGATTTAAC
		dFTN_1326_1.REV	GTGTAGGAATCATACCATCTGCAACCG
		dFTN_13125-26_2.FOR	CTATACTTTCTGATTCCTACACAATATTTATATTCAC
		dFTN_1325-26_2_Sac1.REV	TCAGTAGAGCTCGTGTATCTGCTAAAAAATTAGAGT
		dFTN_1326_Det.For	GCCGATGAAGCTTTACCACT
		dFTN_1325-26_Det.REV	TGCCTGCAGTAATATTCAAAGC
pDMK3-Δ*pdpC*	MNDKYELNIYSDFFKKISS*	dFTN_1319_1_Xho1.FOR	TCAGTACTCGAGCTAAATAACTTTGTGAGCCTTC
		dFTN_1319_1.REV	TTTAAAAAAGTCTGAATAGATATTTAGTTCATATTTGTCG
		dFTN_1319_2.FOR	GAACTAAATATCTATTCAGACTTTTTTAAAAAAATATCGTC
		dFTN_1319_2_Not1.REV	TCAGTAGCGGCCGCTGATAATATCGATGCAATATATGAAA

		dFTN_1319_Det.For	CCAGAATGATTCGGTAGAAAAA
		dFTN_1319_Det.Rev	AAAGGAAAGCAACAGCTCCA
pDMK3-Δ*pdpD*	MDQDINDLLYDTDDLKKEKVR KYRPMIWV*	dFTN_1325_1_Xho1.FOR	TCAGTACTCGAGCACTATCAACTTCTGTAGATCC
		dFTN_1325-26_1.REV	TGTGTAGGAATCAGAAAGTATAGACCAATGATC
		dFTN_13125.FOR	GTCTATACTTTCTTACTTTTTCTTTTTTGAGGTCA
		dFTN_1325_2_Not1.REV	TCAGTAGCGGCCGCCTAAAAATGCAAATATTGATGATATTTATG
		dFTN_1325-26_Det.FOR	GCACCTTTAGCCATTCTTGCT
		dFTN_1325_Det.Rev	AGGAGATATCGCTGCTGGAG
pDMK3-Δ*pdpD/anmK*	LSEYKYCVGIRKYRPMIWV*	dFTN_1325-26_1_Spe1.FOR	TCAGTAACTAGTCACTATCAACTTCTGTAGATCC
		dFTN_1325-26_1.REV	TGTGTAGGAATCAGAAAGTATAGACCAATGATC
		dFTN_13125-26_2.FOR	CTATACTTTCTGATTCCTACACAATATTTATATTCAC
		dFTN_1325-26_2_Sac1.REV	TCAGTAGAGCTCGTGTATCTGCTAAAAAATTAGAGT

dFTN_1325-26_Det.FOR	GCACCTTTAGCCATTCTTGCT
dFTN_1325-26_Det.REV	TGCCTGCAGTAATATTCAAAGC
